# 

**Published:** 1920-04-24

**Authors:** 


### Gifts and Bequests.

The Children's Hospital, Durham, has been left £450 by Mr. J. S. Neilson.

Miss Ellen Atchley, of Clifton, has bequeathed £100 each to the Royal Infirmary and General Hospital, Bristol.

Mr. Robert Barnett, of Edinburgh, left £1,800 among a number of Scottish medical charities, and the residue of his estate to the Edinburgh Royal Infirmary.

The second Earl Brassey, who died from the effects of a taxicab accident, has left £1,000 to East Sussex Hospital, St. Leonards.

Mr. Joseph Braddock, of Oldham, Lancs, left a fortune of the value of £121,826. He bequeathed £1,000 to Oldham Royal Infirmary, and £200 each to the Deaf and Dumb Society and the Workshops for the Blind.

### Nurses Registration Act.

### GENERAL NURSING COUNCIL FOR SCOTLAND.

The members of the General Nursing Council for Scotland, established by the Nurses' Registration (Scotland) Act, 1919, have now been appointed. They are: Captain Charles B. Balfour, C.B., Lord Lieutenant of the County of Berwick (appointed by the Privy Council); Miss Norah Milnes, B.Sc., Director of the School of Social Study and Training, Edinburgh University (appointed by the Scottish Education Department); and the following appointed by the Scottish Board of Health: Dr. A. K. Chalmers, Medical Officer of Health, Glasgow; Dr. Katherine Clark, Assistant Medical Officer to the Edinburgh Education Authority; Dr. H. E. Fraser, Medical Superintendent, Royal Infirmary, Dundee; Col. D. J. Mackintosh, C.B., M.V.O., Superintendent, Western Infirmary, Glasgow; Miss Margaret Bell, Queen's Nurse, Musselburgh; Miss Kathleen L. Burleigh, Matron, Royal Hospital for Sick Children, Edinburgh; Miss Annie Gill, R.R.C., Lady Superintendent of Nurses, Royal Infirmary, Edinburgh; Miss Mary Hunter, Public Health Department, Glasgow; Miss Elizabeth T. Jones, School Nurse, Edinburgh Education Authority; Miss Janet Melrose, R.R.C., Matron, Royal Infirmary, Glasgow; Miss Florence A. Merchant, Matron, Stobhill Hospital, Glasgow; Mr. T. Prentice, Mental Hospital, Hartwood, Lanarkshire; Miss Margaret R. Stewart, Secretary and Treasurer, Scottish Nurses' Club. The Scottish Board of Health intends to convene the first meeting of the Council at an early date.

### Passing Events and Topics.

### The Vaudeville Club.

For the benefit of the Actors' Orphanage, at the King's Hall Theatre, Covent Garden, on Saturday evening, April 24, a special performance of the "Devil's Disciple," by Bernard Shaw, will be given by the Vaudeville Club. The President of this Club and also of the Actors' Orphanage will have a kindly chat with the members prior to the rise of the curtain.

### Great Northern Central Hospital.

Miss Gladys Cooper has kindly promised to organise, at the principal hotels and restaurants, the sale of tickets for the *matinée* at the Palladium on May 14 in aid of the Nurses' Home Section of the Institution's Progress Fund.

### Royal Waterloo Hospital.

A scheme for rebuilding the nurses' home is under contemplation, and the Duchess of Albany is the chairman of a committee with this object, as well as effecting other improvements at an estimated cost of £60,000. Towards this sum £15,900 has been obtained.

### Matrons' and Sisters' Appointments.

Kidderminster Infirmary.—Miss Ada White has been appointed matron. She was trained at St. Bartholomew's Hospital, has been assistant matron at the Reigate and Redhill Hospital and at the General Hospital, Nottingham. Miss White has served at the 1st London General Hospital, Camberwell, and also in France and Belgium, having been sister-in-charge of an ambulance train and sister-in-charge of a casualty clearing-station.

Royal Asylum, Morningside, Edinburgh.—Miss R. E. Appleyard has been appointed assistant matron at the West House. She was trained at Cheddleton Mental Hospital and at North Ormesby Hospital, Middlesbrough; has been sister-in-charge of a house at Alderley Edge Epileptic Colony, night superintendent at Melrose District Asylum, and has done private nursing.

Children's Hospital, Royal Infirmary, Sunderland.—Miss Sarah G. I. Bainbridge has been appointed matron. She was trained at the Royal Infirmary, Bradford, where she was later casualty and out-patient sister, theatre, and night sister. She has also been assistant matron of Rawdon Auxiliary Hospital, and of the Royal Aberdeen Hospital for Sick Children, Aberdeen.

Royal Aberdeen Hospital for Sick Children.—Miss Gertrude Moggach has been appointed assistant matron. She was trained at the Bradford Royal Infirmary, and was later ward and night sister there. She has also held the post of theatre sister in France and Belgium, sister at the West London Hospital, and night sister at the Royal Aberdeen Hospital for Sick Children. Miss Moggach holds the certificate of the Central Midwives Board.

Shirley Warren Infirmary, Southampton.—Miss Mary Farmer has been appointed home sister. She was trained at the Woolwich Union Infirmary, has been night sister at the Taunton and Somerset Hospital, and matron of the Lysaght Military Hospital, Burnham-on-Sea.

Bolton Infirmary and Dispensary.—Miss Ruth Hall has been appointed theatre sister. She was trained at the North Riding Infirmary, Middlesbrough, and has since been theatre sister there and at the Military Hospital, Stourbridge.

Royal Hospital, Chesterfield.—Miss F. C. Tomkins has been appointed night sister. She was trained at the Stanley Hospital, Liverpool, has been staff nurse at the Hospital for Women, Soho Square, sister at the Royal Infirmary, Wigan, the Broadstairs Auxiliary Hospital, Liverpool, and in the Royal Naval Nursing Service Reserve.

Edinburgh Royal Maternity and Simpson Memorial Hospital.—Miss Agnes Roger Bett has been appointed matron. She was trained at Edinburgh Royal Infirmary and Edinburgh Royal Maternity Hospital, where she was later sister. She has lately been matron of Birmingham Maternity Hospital.

### Forthcoming Events.

East London Hospital for Children, Shadwell, E.—General Court of Governors, Monday, April 26, 3 p.m.

Invalid Children's Aid Association.—By kind permission of the Marquis and Marchioness of Londonderry, the annual meeting will be held at Londonderry House, Park Lane, W., on Tuesday, April 27, at 3 p.m. The American Ambassador will preside, and the Lord Bishop of Stepney, Miss Irene Vanbrugh, Major Souttar, C.B.E., F.R.C.S., Major Beith (Ian Hay), and Gerald du Maurier will be among the speakers. Admission by ticket only, on application to the Secretary, 69 Denison House, 296 Vauxhall Bridge Road, Westminster, S.W.

United Kingdom Conference on the Prevention of Diseases of the Teeth.—Manchester, May 13-15, 1920. Programme, with full particulars, from the Food Education Society, Danes Inn House, 265 Strand, W.C. 2, sending 1½d. stamp.

### The College of Nursing (Limited by Guarantee).

### EDINBURGH CENTRE.

Owing to the unavoidable absence of Dr. Sym from Edinburgh, the lecture on "Eye Diseases from a Nursing Point of View," announced for Thursday, April 29, has been postponed till Thursday, May 13, when it will be given by him in the Nurses' Club, 8 Drumsheugh Gardens, at 3.30 p.m.

### BIRMINGHAM CENTRE.

Annual Meeting.

The Annual Meeting of the Birmingham Centre was held in the Lecture Theatre of the General Hospital on April 14. Miss Musson, R.R.C., was in the chair. After the usual business had been transacted and the hon. officers re-elected, Miss Sparshott, O.B.E., R.R.C., spoke upon "The Aims and Endeavours of the College," and her convincing address was closely followed and appreciated. At the conclusion of the address a discussion ensued as to the advisability of a 56-hours working week for nurses. Later tea was served in the Board Room.

Subscriptions.

Members are reminded that subscriptions for the current year are now due and should be forwarded to the Hon. Treasurer, Mrs. Boeddeeker, Ellerslie, Ascot Road, Moseley.

Forthcoming Lecture.

Dr. Thomas Wilson will give a post-graduate lecture on "Gynæcology" on Tuesday, April 27, at 5.30 p.m., in the Lecture Theatre of the General Hospital, Birmingham. Entrance fee: Members, free; non-members, 1s.

### LIVERPOOL CENTRE.

Annual Meeting.

The Annual Meeting will be held on Wednesday, April 28, at 6 p.m., at the Royal Infirmary. Miss Cummins, R.R.C., has kindly invited the members to tea. It is hoped that all members will make a special effort to be present at this meeting.

Subscriptions.

Subscriptions 1920-21 are now due, and the Treasurer, Miss Golding, 11 Hargreaves Road, Sefton Park, will be pleased to receive them as soon as possible.

The lecture given on the 14th inst. by C. B. Alexander, Esq., M.R.C.S., L.R.C.P., on "Orthopædics," was very well attended. The lecturer's practical demonstrations and the fact that the lecture was held in the new and up-to-date Orthopædic Department were greatly appreciated.

